# Robust Porous TiN Layer for Improved Oxygen Evolution Reaction Performance

**DOI:** 10.3390/ma15217602

**Published:** 2022-10-29

**Authors:** Gaoyang Liu, Faguo Hou, Xindong Wang, Baizeng Fang

**Affiliations:** 1 State Key Laboratory of Advanced Metallurgy, University of Science and Technology Beijing, Beijing 100083, China; 2 School of Metallurgical and Ecological Engineering, University of Science and Technology Beijing, Beijing 100083, China; 3 Department of Chemical and Biological Engineering, University of British Columbia, 2360 East Mall, Vancouver, BC V6T 1Z3, Canada

**Keywords:** proton exchange membrane water electrolysis, oxygen evolution reaction, iridium oxide, titanium nitride, thermal nitriding

## Abstract

The poor reversibility and slow reaction kinetics of catalytic materials seriously hinder the industrialization process of proton exchange membrane (PEM) water electrolysis. It is necessary to develop high-performance and low-cost electrocatalysts to reduce the loss of reaction kinetics. In this study, a novel catalyst support featured with porous surface structure and good electronic conductivity was successfully prepared by surface modification via a thermal nitriding method under ammonia atmosphere. The morphology and composition characterization-confirmed that a TiN layer with granular porous structure and internal pore-like defects was established on the Ti sheet. Meanwhile, the conductivity measurements showed that the in-plane electronic conductivity of the as-developed material increased significantly to 120.8 S cm^−1^. After IrO_x_ was loaded on the prepared TiN-Ti support, better dispersion of the active phase IrO_x_, lower ohmic resistance, and faster charge transfer resistance were verified, and accordingly, more accessible catalytic active sites on the catalytic interface were developed as revealed by the electrochemical characterizations. Compared with the IrO_x_/Ti, the as-obtained IrO_x_/TiN-Ti catalyst demonstrated remarkable electrocatalytic activity ($\eta_{{10\;\mathrm{mA}\;\mathrm{cm}}^{-2}}\;$= 302 mV) and superior stability (overpotential degradation rate: 0.067 mV h^−1^) probably due to the enhanced mass adsorption and transport, good dispersion of the supported active phase IrO_x_, increased electronic conductivity and improved corrosion resistance provided by the TiN-Ti support.

## 1. Introduction

Hydrogen is a green energy carrier which can be sustainably produced by various strategies such as photocatalysis [1,2,3,4,5,6] and electrocatalysis [7,8,9]. Amongst the diverse hydrogen production approaches, especially compared with the alkaline water electrolysis [10], proton exchange membrane (PEM) water electrolysis has the advantages of fast start, high current densities and energy efficiency, and low gas crossover, and is thus considered to be the most promising technology for electrocatalytic hydrogen production [11]. The electrolytic splitting of water involves two half reactions including hydrogen evolution reaction (HER) and oxygen evolution reaction (OER), and the slow kinetic process of OER on the surface of the anode limits greatly the performance improvement of PEM water electrolysis [12]. In order to compensate for the loss of electrode kinetics, PEM water electrolysis generally uses precious metals (e.g., Ir- and Ru-based materials) as catalysts, and the expensive price of catalysts is also one of the important factors restricting the development of PEM water electrolysis [13]. In order to reduce energy consumption and improve electrode performance, electrocatalysts with high catalytic activity and stable electrochemical properties are highly required.

A potential way to improve the electrocatalytic activity of OER and reduce the usage of noble metals is to load the catalytic active phase on the support [14,15]. The biggest advantage of supported catalysts is that the good dispersion of the active component can be achieved by improving the microstructure of the support material itself, thereby increasing the catalytic activity area. Meanwhile, it was reported the interactive effect between the conductive support and active phases may also accelerate the catalytic activity [16,17,18]. Currently, due to the corrosion caused by the high overpotential and oxygen evolution environment, ordinary carbon supports are no longer applicable. Ideal supports are required to have good corrosion resistance, good conductivity and good binding force with the active components. Increasing the potential of the OER and the oxygen-rich environment will lead to corrosion loss of the support materials; therefore, the choice of supports material can only be limited to some corrosion-resistant oxides or ceramic materials. At present, the supports under investigation mainly include SiC-Si [16] with low electron conductivity, TinO_2n−1_ [17] antimony-doped tin oxide (ATO) [18] and TiC [19], etc. It was reported that with the improvement of the electron conductivity of the support, even though the loading proportion of the active component decreased from 90 wt% (SiC-Si) to 20 wt% (TinO_2n−1_, ATO and TiC), and the supported catalyst still has an OER electrocatalytic activity comparable to that of the pure active component.

Other than the nanoparticle structured support, various nano- and porous structured supports have been widely researched, e.g., three-dimensional ordered macropore (3-DOM) [20], nanowires [21], etc. Compared with the nanoparticle support, the electrocatalytic activity was significantly improved due to the enhancement of the catalytic activity area and the better gas-liquid transport channel provided by the porous structure [15]. However, there are still many problems when utilizing these catalysts for electrode preparation. A crushing ultrasonic process could destroy the original microstructures. In addition, the additives of the ionomer and binder which are gas proof and electronic insulated material will lead to the poor electronic conductivity and bad mass diffusion. Recently, researchers have devoted time to developing porous matrix materials with high exposure of a large active surface area, a high electronic conductivity, and a good corrosion resistance, which are favorable for the OER. Various nanostructured supports, such as ordered porous layer [22] array [23,24], cross-linked nanowires [25], etc. have been established on carbon paper (CP), carbon cloth (CC), Ti felt, etc., and the as-obtained supports were then used to load the active phase as the integrated porous electrode [26,27]. On the one hand, the microstructure of the synthesized integrated porous electrode can be maintained and can contribute to the improved active area as well as fast mass transport. On the other hand, it avoids the usage of ionomer, which is conducive to electron transport and gas-liquid transport. It can be expected that the dispersion of the active phases, the electronic conductivity as well as the porous structure of the support have an important impact on the catalytic activity of the catalyst for the OER. Therefore, it is necessary to design catalysts from the surface composition and the microstructure regulation of the support by the optimization of the preparation method.

Recently, titanium and its alloys have been widely used to produce the bipolar plates (BPs) and the liquid and gas diffusion layer (LGDL) due to their features of excellent electronic conductivity and corrosion resistance, and they are also believed to be a potential electrocatalytic matrix material [28,29]. However, a big issue of titanium and its alloys is that a passive film with poor conductivity forms in the working environment and leads to the reduction in performance and durability [30].

The nitriding of the titanium surface has been proved to effectively reduce the contact resistance of the electrode. Especially, the nitriding under ammonia as a nitrogen source could obtain a high-quality (low oxygen, high conductivity, corrosion resistance) titanium nitride surface [31,32]. Meanwhile, the strong alkalinity and high reducibility of ammonia may have an impact on the microstructure and porosity of titanium nitride surface, which contributes to the dispersion of the active phases and thus improved active surface area [33]. In this study, the modifications of the surface composition (titanium nitride coating) as well as the surface microstructure (porous structure) of the titanium support were realized via a modified thermal nitriding method under ammonia environment. Then, the titanium nitride coated support was further loaded with the active components to fabricate an integrated porous electrode towards the OER on anode in PEM water electrolysis. From the composition and microstructure aspects, the use of the novel support not only optimizes the dispersion of the active phases, the charge as well as the mass transfer process of the OER, and finally enhances the electrocatalytic activity, but also greatly reduces the amount of noble metal used, and effectively decreases the cost.

## 2. Experimental Section

### 2.1. Materials

The commercial titanium alloy (Ti-6Al-4V) sheet was purchased from Xiamen Tmax Battery Equipments Limited, China. Nafion^®^ perfluorinated resin solution (5 wt%) was purchased from Sigma-Aldrich (Shanghai, China). Iridium chloride acid (H_2_IrCl_6_·H_2_O) and IrO_2_ powders were acquired from Alfa Aesar (Shanghai, China). All the other chemicals used in the present study were purchased from Sinopharm Chemical Reagent Beijing Co., Ltd. (Beijing, China), and used as-received without further purification.

### 2.2. Synthesis of TiN and IrO_x_/Ti

Pretreatment of the Ti sheet [34]: First, a 1 mm thick Ti sheet was cut into small pieces with a size of 10 mm × 10 mm, and then the samples were polished with 400#, 800#, 1000#, 1500# grit paper (SiC) and polishing cloth, sequentially. Next, the samples were washed by ultra-sonication in acetone, ethanol and deionized (DI) water for 30 min each time, and then dried under vacuum at 70 °C.

Nitriding of the Ti sheet: NH_3_ gas was applied as the N source. Instead of using stainless steel foil, the pretreated samples were placed in a corundum crucible. Before the nitriding experiment, the crucible was placed in a drying oven and then transferred into a tube furnace filled with high-purity N_2_ atmosphere for 1 h. The nitriding process was carried out in the tube furnace filled with high-purity NH_3_ for 1 h at room temperature followed by heating to 600 °C at a heating rate of 5 °C min^−1^, and nitriding for 2 h. The samples were taken out after cooling to ambient temperature. The obtained nitrided Ti sheet was marked as TiN-Ti.

Synthesis of IrO_x_/TiN-Ti [25]: to load IrO_x_ on the nitrided Ti (TiN) sheet, 500 mg of H_2_IrCl_6_·H_2_O was firstly added to 3 mL of methanol solution, and the solution was sonicated for 1 h. Then, a piece of dried TiN sheet (1 × 1 cm^2^) was soaked in the H_2_IrCl_6_·H_2_O solution for 30 min to obtain H_2_IrCl_6_·H_2_O/TiN. Afterwards, H_2_IrCl_6_·H_2_O/TiN-Ti was thermally annealed at 500 °C for 30 min in air. The loading density of IrO_x_ on the TiN-Ti could be adjusted by repeating the above-mentioned soaking-annealing processes, and the final loading of IrO_x_ was ca. 0.2 mg cm^−2^. For comparison, the electrode using the un-nitrided Ti sheet as a support was prepared according to the procedures similar to those described above. The obtained IrO_x_ supported on the nitriding Ti sheet and the n-nitriding Ti sheet was marked as IrO_x_/TiN-Ti and IrO_x_/Ti, respectively.

### 2.3. Physiochemical Characterizations

The crystalline structures of the prepared samples were characterized by X-ray diffraction (XRD) using a Marcogroup diffractometer (MXP21 VAHF) with a Cu-Kα radiation source (λ = 1.54056 Å) to characterize catalyst crystalline structure. The morphology, particle size and composition information of samples were studied using Scanning electron microscopy (ZEISS and LEO-1530 FESEM) with EDS. The surface chemical states of the as-synthesized samples were studied by X-ray photoelectron spectroscopy (XPS) (Kratos AXIS Ultra DLD).

### 2.4. Electrochemical Characterizations

All the electrocatalytic tests were carried out in a three-electrode configuration in 0.5 M H_2_SO_4_ electrolyte solution at room temperature using an energylab XM electrochemical workstation. The as-synthesized IrO_x_/TiN-Ti and IrO_x_/Ti were directly used as the working electrode (WE). A 10 × 10 mm platinum plate was used as the counter electrode (CE), and Ag/AgCl (saturated) was used as the reference electrode (RE). The Ag/AgCl (saturated) reference was calibrated prior to each measurement in Ar/H_2_-saturated 0.5 M H_2_SO_4_ solution using a clean Pt wire as the working electrode. In addition, prior to the electrochemical measurements, the electrochemical cell was purged with nitrogen to completely remove the air in the electrolyte. All electrochemical tests were performed at room temperature. All potentials in this paper were converted to reversible hydrogen electrode (RHE) reference potential according to literature [22]. For the commercial IrO_2_ powder, the catalyst ink was prepared by ultrasonic treatment of the mixture of the electrocatalyst (1 mg), ethanol (5 mL) and Nafion (5 wt%, 50 μL) in an ice bath for 2 h. The working electrode (IrO_2_ loading was about 0.2 mg/cm^2^) was made by casting uniformly dispersed catalyst ink onto a glassy carbon electrode (area 0.283 cm^2^) and being dried in air.

The details for the test protocol are as follows. First, the potentiostatic electrochemical impedance spectroscopy (PEIS) was measured at open circuit voltage (OCV) after the system was stable. Then, 50 cycles of cyclic voltammetry (CV) were recorded from 0 to 1 V (vs. RHE) at a scan rate of 50 mV/s to stabilize the OER performance of the electrocatalyst. Next, the electrochemically active area (ECSA) was obtained by CV tests at different scanning speeds (3, 5, 7, 10, 20, 50, 100, 200, 300 mV s^−1^) in the non-Faraday potential range (OCV ± 50 mV). After that, the linear scan voltammetry (LSV) tests were carried out in a range of 1.2–1.6 V vs. RHE. Finally, the PEIS at 1.53 V vs. RHE with amplitude of 5 mV was carried out. The stability of catalysts was assessed at a constant current density of 10 mA cm^−2^ using chronopotentiometry (CP).

## 3. Results and Discussion

**Figure 1:**
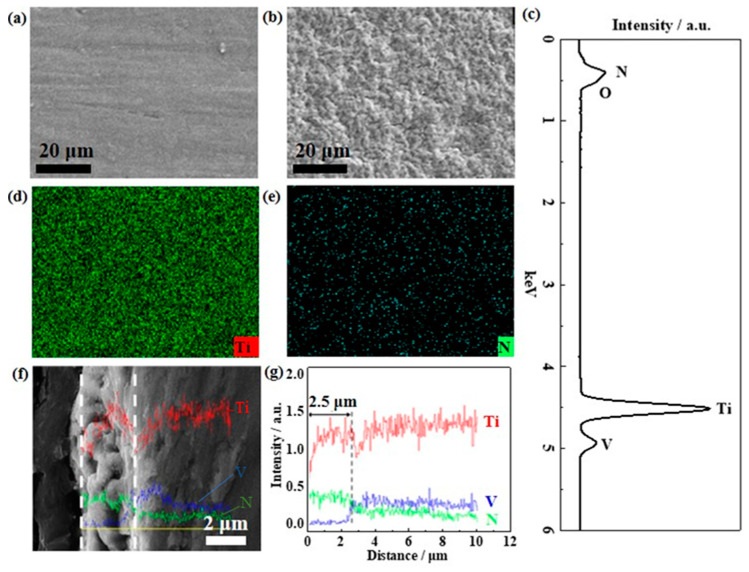
SEM images: (**a**) Ti sheet after the mechanical polishing, (**b**) TiN-Ti. (**c**) EDS spectra of the TiN-Ti, EDS elemental mappings of the TiN-Ti: (**d**) Ti, (**e**) N; (**f**) Cross-section SEM image and (**g**) EDS elemental liner scan of the TiN-Ti.

Figure 1a,b show the SEM images of the pretreated Ti sheet before and after the thermal nitriding under NH_3_ atmosphere. It can be seen that the Ti sheet that has not been nitrided appears as a flat and smooth surface, and there are still scratches left by the mechanical polishing. No obvious defects and porous structures were observed. The scratches on the surface of the Ti sheet after the nitriding under NH_3_ atmosphere have been completely covered by the nitride coating. In addition, the surface of the coating after nitriding is granular porous structure mainly due to the strong alkalinity and high reducibility of NH_3_. Meanwhile, the high temperature thermal expansion can lead to rich internal pore-like defects between the particles, but no obvious gaps and cracks appeared. The EDS spectra shown in Figure 1c reveal that the elements present in the nitriding sample are mainly Ti, V and N. From the EDS elemental mappings of the TiN-Ti, both the Ti and N are uniformly distributed, but the specific phases in which they exist need to be further analyzed. Figure 1f,g show the cross-section SEM image and the corresponding EDS liner scans after the thermal nitriding. The results showed that there were significant pore-like defects inside the coating with a thickness of about 2.5 μm. The presence of porous morphology and defects will help to disperse the active components when used as the supporting material, which may significantly enhance the adhesion of the active phases and also increase the number of active sites that can catalyze the OER process [35].

**Figure 2:**
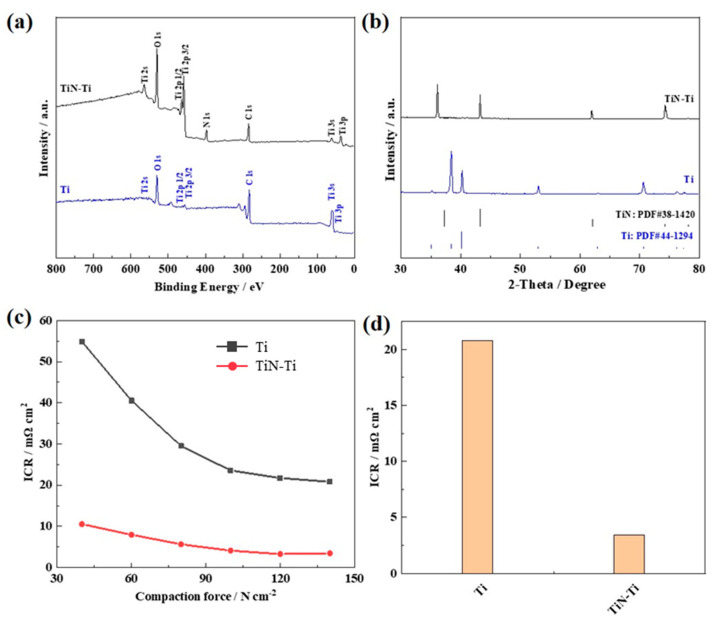
(**a**) XPS survey spectra, and (**b**) XRD patterns of the Ti sheet and TiN-Ti, (**c**) Relationship between the ICR and the compaction force, (**d**) ICR at 140 N cm^−2^ of the Ti and the TiN-Ti.

The surface chemical states of the Ti sheets before and after the thermal nitriding under NH_3_ atmosphere were further investigated using X-ray photoelectron spectroscopy (XPS). The XPS survey spectra in Figure 2a confirm the presence of the corresponding N element in the TiN/Ti, but no N element in the Ti sheet. It indicated that the successful nitriding of the Ti surface. Figure 2b shows the XRD patterns of the Ti sheets before and after the thermal nitriding. The phase composition of the Ti sheet is mainly α-Ti (pdf#44-1294) [36], while the nitrided sample has a new phase, TiN (pdf#38-1420) [37]. In the XRD patterns, two distinct diffraction peaks were found at diffraction angles of 36.7° and 42.7°, corresponding to the (111) and (200) facets of TiN, respectively. Therefore, the SEM, EDS, XPS and XRD results confirmed that a TiN layer with rich granular porous structure and internal pore-like defects was successfully established on the Ti sheet. Meanwhile, the conductivity measurements by the four-probe method showed that the in-plane electronic conductivity increased from 2.6 S cm^−1^ of the unmodified Ti sheet to 120.8 S cm^−1^ of the Ti sheet after the thermal nitriding. The improved in-plane electronic conductivity of the TiN-Ti was further confirmed with the ICR measurement as reported by Wang et al. As it is shown in Figure 2c,d, the ICR of the TiN-Ti is significantly decreased to 3.4 mΩ cm^2^, and lower than the unmodified Ti sheet (20.8 mΩ cm^2^). It should be noted that even though the unmodified Ti sheet is pretreated as illustrated in the experimental part, there still could be an oxidized TiO_x_ layer, which would result in lower in-plane electronic conductivity. The improved in-plane electronic conductivity of the TiN-Ti can be ascribed to the formation of the highly conductive TiN phase. Overall, both the promoted porous structure and enhanced electronic conductivity may contribute to more accessible active sites when the TiN-Ti is used as a support to load the active phases.

**Figure 3:**
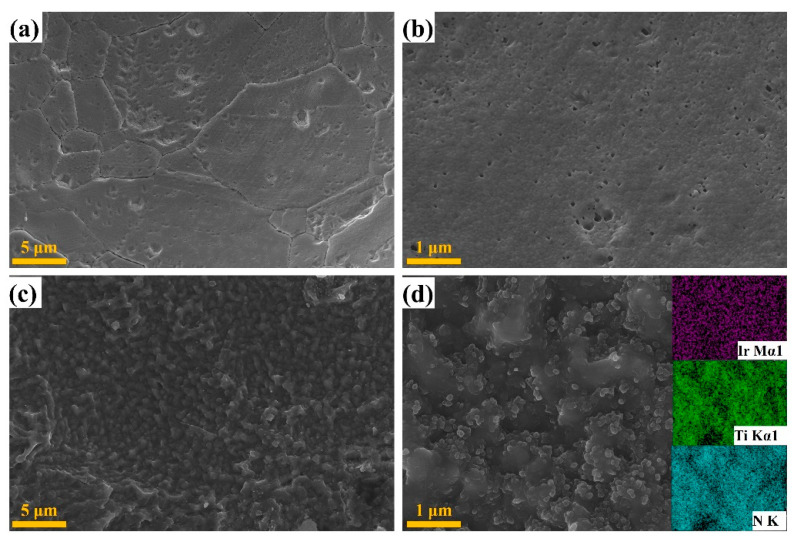
SEM images: (**a**,**b**) IrO_x_/Ti, (**c**,**d**) IrO_x_/TiN-Ti, and the inset of (**d**) is the EDS elemental mapping images of the TiN-Ti.

Figure 3a–d show the SEM images of the prepared IrO_x_/Ti and IrO_x_/TiN-Ti, respectively. It can be seen that catalyst layers were successfully loaded on both the Ti sheet and TiN-Ti with the soaking-annealing process, and the morphology of the IrO_x_/Ti and IrO_x_/TiN-Ti did not markedly alter after loading IrO*_x_* nanoparticles. For the IrO_x_/Ti, it can be seen from Figure 3a,b that the surface remained flat but cracks and pits appeared, and it can be associated with the generated gas during the pyrolysis reaction of IrO_x_. While for the IrO_x_/TiN-Ti as shown in Figure 3c,d, the porous structure and rough surface can still be maintained and IrO_x_ nanoparticles with a typical diameter of around 80–100 nm are uniformly distributed on the TiN-Ti surface. Even though the porous structure decreased after loading IrO_x_ during the soaking-annealing process, the porous structure of the TiN-Ti could result in completely different morphology of the catalytic layers. It is believed that better dispersion of IrO_x_ can be achieved with the TiN-Ti, and thus better ECSA and catalytic activity. The insert in Figure 3d presents the elemental mapping images and they illustrated that Ir is distributed uniformly on the TiN support with a very high density. It can be deduced that the TiN-Ti support can effectively promote the dispersion of IrO_x_ than the un-nitrided Ti sheet.

**Figure 4:**
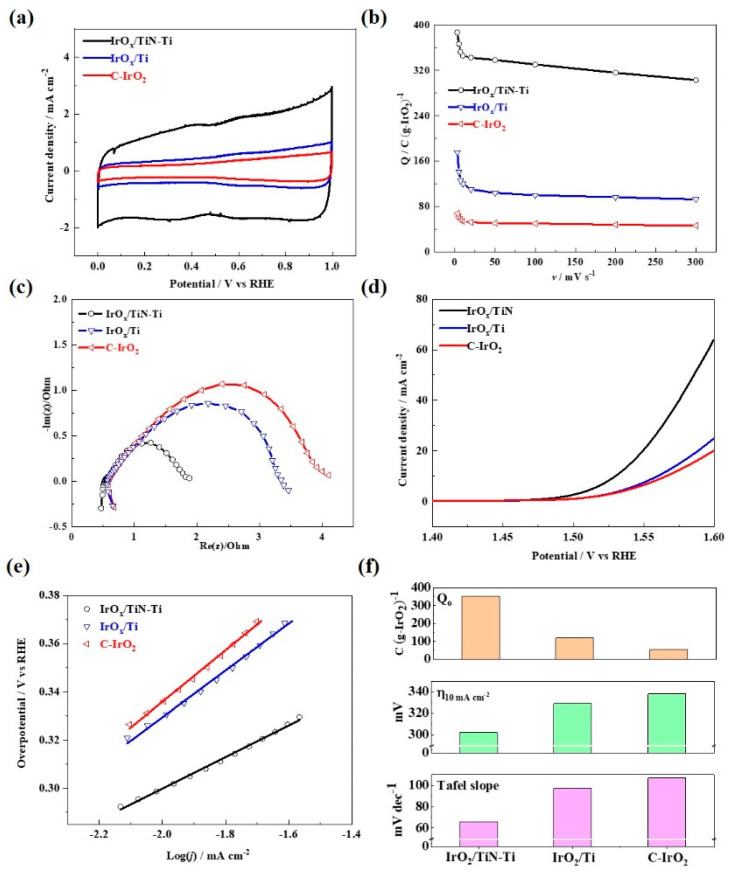
(**a**) Representative CVs recorded with a scan rate of 20 mV s^−1^, (**b**) The plots of the charge as a function of the scan rate, (**c**) Nyquist diagram measured at 1.53 V vs. RHE during oxygen evolution, the inset is the equivalent circuit, (**d**) iR-corrected steady-state polarization curves, (**e**) Tafel plots and (**f**) Comparison of Q_o_, $\eta_{{10\;\mathrm{mA}\;\mathrm{cm}}^{-2}}$, Tafel slopes of the IrO_x_/TiN-Ti, IrO_x_/Ti and C-IrO_2_. All the electrochemical measurements were tested in 0.5 mol L^−1^ H_2_SO_4_ at 25 °C.

In order to evaluate the effect of different supports on the catalytic activity of the active phase towards the OER, the specific activity based on the same IrO_x_ loading was used in the following discussion. The three-electrode test was carried out in 0.5 mol L^−1^ H_2_SO_4_ at 25 °C. The working electrode was the prepared IrO_x_/Ti, IrO_x_/TiN-Ti, and GC supported commercial IrO_2_ (marked as C-IrO_2_), and the CV scan was carried out in the double layer potential window range (0–1 V vs. RHE) and the CV test results are presented in Figure 4a. It can be seen that all electrodes showed a similar IrO_2_ redox process, i.e., there are two pairs of redox peaks. The redox peaks located near 0.6 V correspond to Ir_III_/Ir_IV_ redox pairs, and redox peaks located near 0.95 V correspond to Ir_IV_/Ir_VI_ redox pairs [38]. The catalytic activity area can be evaluated by integrating the voltammogram in the anode part of the CV curve obtained at a scanning speed of 20 mV s^−1^, and the calculated voltametric charges are shown in Table 1, and they were considered to be associated with the number of active sites or the catalytic active area [39]. It shows that the voltametric charges of the IrO_x_/TiN-Ti are greater than that of the IrO_x_/Ti, which can be attributed to the rich porous structure and internal pore-like defects possessed by the IrO_x_/TiN-Ti, which can form a good dispersion of the IrO_x_ nanoparticles.

Figure 4b shows the voltametric charges for the C-IrO_2_ and the supported catalysts obtained at different potential scan rates. It could be seen that the charges show a significant decrease trend as the sweep speed increases, which is due to the hysteresis of charge transfer or the relatively slow transfer speed [38]. At high sweep speeds, charge transfer can only occur at the active interface (“outer” surface) in direct contact with the electrolyte, corresponding to the outer surface charge (Q_o_); At low sweep speed, proton exchange energy occurs at the active interface of the entire electrode (the “internal” interface and the “outside” surface of the proton conduction) slowly, corresponding to the total electrode charge (Q_t_) [18,21]. Table 1 and Figure 4f list the calculated Q_o_ and Q_t_ of the synthetic electrodes according to literature. Compared with the IrO_x_/Ti, both the Q_o_ and Q_t_ have been significantly improved, indicating that the unique porous structure and internal pore-like defects in the TiN-Ti promote the dispersion of the active components, as well as the accessibility of the catalytic sites. In addition, according to the literature [40,41,42,43,44,45], the presence of a porous structure with abundant macropores (or mesopores with a large pore size) in the catalyst support (i.e., TiN-Ti) facilitates fast mass transport, resulting in improved electrocatalytic performance.

Figure 4c shows the EIS behavior of the different electrodes, and the simulation data results obtained using the equivalent circuit in the insert are listed in Table 1. As shown in Table 1, compared with the IrO_x_/Ti and the C-Ti, the *R*_Ω_ of the IrO_x_/TiN-Ti is significantly decreased, which is mainly due to the fact that the conductivity of the TiN-Ti is lower than that of the unmodified Ti sheet. It has been reported that the Ti surfaces can be easily oxidized and the passive film with poor conductivity could be formed in the anodic working environment [32,37]. For the different electrodes, it can be seen that compared with the C-IrO_2_, the *R*_ct_ decreased for both the supported electrodes IrO_x_/Ti and IrO_x_/TiN-Ti, indicating the charge transfer has been promoted due to the interactive effect between the support and active phases [17,18]. Meanwhile, the lowest *R*_ct_ was obtained with the IrO_x_/TiN-Ti. It can be deduced that both the optimized microstructure and the enhanced electronic conductivity of the porous TiN layer are conducive to the charge transfer process at the heterogeneous catalytic interface.

Steady state polarization curves of the three prepared electrodes were recorded in the potential region for the OER (1.4–1.6 V vs. RHE) as shown in Figure 4d. At a current density of 10 mA cm^−2^, the overpotentials of the IrO_x_/TiN-Ti, IrO_x_/Ti, and C-IrO_2_ were 302 mV, 329 mV, and 338 mV, respectively (Figure 4f). Compared with the recently reported electrocatalysts towards the OER, the IrO_x_/TiN-Ti in this study shows remarkable catalytic activity [46,47,48]. The Tafel slope reflects the reaction mechanism of the OER process, and it is generally used to evaluate reaction kinetics of electro-catalytic materials. Figure 4e,f show the Tafel curves for the different electrodes. Generally, the IrO_x_/TiN-Ti exhibited better kinetic parameters than the IrO_x_/Ti and C-IrO_2_. The IrO_x_/TiN-Ti shows the smallest Tafel slopes (ca. 66 mV dec^−1^). It suggests the reaction mechanism due to the adsorption contribution of intermediates. Overall, the loading of IrO_x_ nanoparticles on the TiN-Ti could accelerate the reaction kinetics, and thus remarkably enhance the catalytic activity.

**Figure 5:**
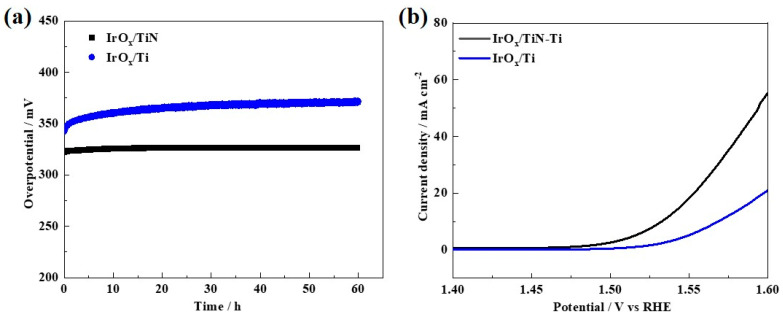
(**a**) Chronopotentiometric curves of the IrO_x_/TiN-Ti and IrO_x_/Ti recorded at a constant current density of 10 mA cm^−2^. (**b**) iR-corrected steady-state polarization curves of the IrO_x_/TiN-Ti and IrO_x_/Ti after 60 h of OER.

The stability is a key challenge for electrocatalytic materials to meet the demanding targets of practical applications in PEM water electrolyzers [49]. The electrochemical stability of the prepared electrodes was evaluated by chronopotentiometric (CP) method at a constant current density of 10 mA cm^−2^ in 0.5 M H_2_SO_4_ electrolyte. The results are shown in Figure 5a.

During the first 10 h of stability testing, the overpotential required to achieve current densities of 10 mA cm^−2^ increases significantly for both the IrO_x_/Ti and IrO_x_/TiN-Ti mainly due to the bubble effect, which reduced the catalyst utilization. After this stable period, the IrO_x_/TiN-Ti shows negligible degradation and good stability until 60 h, and the overpotential required to achieve current density of 10 mA cm^−2^ slightly increases from 322 mV to 326 mV. The whole process of the overpotential degradation rate was only 0.067 mV h^−1^, which is much smaller than that of the IrO_x_/Ti (from 342 mV to 371 mV, and the overpotential degradation rate was 0.483 mV h^−1^). It indicated that the IrO_x_/TiN-Ti can continue to operate stably without a sharp increase. Figure 5b presents the steady-state polarization curves of the IrO_x_/TiN-Ti and IrO_x_/Ti after 60 h of the OER. Compared with Figure 4d, there is less attenuation of catalytic activity before and after the 60 h stability test for the IrO_x_/TiN-Ti than IrO_x_/Ti. Overall, the IrO_x_/TiN-Ti has been proved to show remarkable electrocatalytic activity and superior stability. The application of support materials with rich porous microstructure and the enhanced surface conductivity could have important consequences for PEM water electrolysis technology and production of green hydrogen.

## 4. Conclusions

In this study, a TiN layer with rich granular porous structure and internal pore-like defects was successfully established on the Ti sheet via the thermal nitriding method under NH_3_ atmosphere. The strong alkalinity and high reducibility of NH_3_ not only result in porous microstructured surfaces, but also lead to a high-quality TiN layer with sufficient electronic conductivity. All these features were characterized and verified by SEM, EDS, XPS and XRD analyses. Furthermore, the novel TiN-Ti was used as a support for loading IrO_x_ active phases towards the OER. The electrochemical tests revealed that significant enhancement of the OER activity was obtained for the IrO_x_/TiN-Ti compared with the un-nitrided TiN supported catalysts. The remarkable catalytic activity is ascribed to the more accessible active sites created as well as the faster charge transfer on the catalytic interfaces, which are mainly due to the good dispersion, and high electronic conductivity provided by the porous structured TiN layer. The IrO_x_/TiN-Ti also showed good durability under a current density of 10 mA cm^−2^ in a period of 60 h due to the high corrosion resistance of the TiN.

## Figures and Tables

**Table 1:** Summary of electrochemical characterization of different electrodes.

	CV			EIS		Tafel Slopes
	Q ^(a)^	Q_t_ ^(a)^	Q_o_ ^(a)^	R_Ω_ ^(b)^	R_ct_ ^(b)^	S1 ^(c)^
IrO_x_/TiN-Ti	340	351	303	0.493	1.331	66
IrO_x_/Ti	112	117	96	0.562	2.781	97
C-IrO_2_	52	53	37	0.604	3.330	107

Notes: ^(a)^ The anodic charge (Q/mC cm^−2^), total charge (Q_t_/mC cm^−2^), outer charge (Q_o_/mC cm^−2^) of all prepared electrodes calculated from the cyclic voltammograms. ^(b)^ Ohmic resistance (*R*_Ω_/ohm cm^2^), charge-transfer resistance (*R*_ct_/ohm cm^2^). ^(c)^ Tafel slopes (mV dec^−1^).

## Data Availability

Not applicable.
